# Acoustic variation of spider monkey (*Ateles geoffroyi*) contact calls is related to caller isolation and affects listeners’ responses

**DOI:** 10.1371/journal.pone.0213914

**Published:** 2019-04-03

**Authors:** José D. Ordóñez-Gómez, Ana M. Santillan-Doherty, Kurt Hammerschmidt

**Affiliations:** 1 Cognitive Ethology Laboratory, German Primate Center, Göttingen, Germany; 2 Neurociencias, Instituto Nacional de Psiquiatría Ramón de la Fuente Muñiz, Mexico City, Mexico; McGill University, CANADA

## Abstract

Group living animals produce vocalizations denominated “contact calls” to maintain contact with out-of-sight group members. These calls have been shown to vary with caller identity and distance to potential listeners. However, it is not clear whether the acoustic variation of contact calls is related to caller social isolation (e.g., inside or outside a subgroup) and listeners’ responses that can be helpful to maintain contact. Here, we addressed these questions in spider monkeys (*Ateles geoffroyi*), a Neotropical primate that exchanges contact calls denominated “whinnies”, which show graded variation related to caller immediate behavior and distance between callers. Using 566 whinnies produced by 35 free-ranging adult spider monkeys recorded at ≤ 20 m from microphones, we first analyzed whether the acoustic variation of spontaneous whinnies (i.e., whinnies that are not responses to previous whinnies) is related to caller social isolation or whether acoustic variation is related to the likelihood of eliciting a response whinny from another individual. Secondly, we assessed whether listeners’ responses (i.e., time to respond vocally, acoustic characteristics of response whinnies, orienting behaviors) were related to the acoustic variation of previous whinnies. Our study revealed that callers that were outside a subgroup produced whinnies with a lower fundamental frequency (F0), which travels longer distances, and increases the likelihood of producing a response whinny. Moreover, listeners (i.e., responders) responded faster to lower F0 whinnies. However, the acoustic variation (i.e., F0 variation) in response whinnies was better explained by the separation distance between callers, than by the acoustic variation of the previous whinny. Overall, our results suggest that whinny variation facilitates vocal contact to callers that are outside a subgroup, and that context and whinny variation affect listeners’ responses.

## Introduction

Group living animals establish social relations that serve to defend their territory and share food resources (e.g., [1]). Due to several factors, such as vegetation density and social dynamics, group members may lose visual contact with each other. Using vocalizations often termed “contact calls”, animals can keep contact with group members who may be out of sight [2–4]. Contact calls have been shown to vary in relation to caller identity and distance to presumed listeners [5–7]. Moreover, playback experiments revealed that individuals that most commonly respond to the speaker are those close-associates of the individual whose calls are broadcasted (e.g., [3, 8, 9]). However, it is less clear whether the acoustic variation of contact calls facilitates vocal contact in contexts in which callers are outside their group or prompts responses in conspecifics that facilitate contact (orienting behaviors e.g., looking towards the caller, scanning).

Studies with different call types (e.g., alarm and aggressive calls) showed that call acoustic variation can be related to listeners’ responses (e.g., [10, 11]). Different ideas have been put forward to explain these relationships. On the one hand, it has been suggested that calls with arousing acoustic features can elicit innate responses in listeners [4, 12]. For instance, it is proposed that abrupt onsets and chaotic spectral structures can capture listeners’ attention through the activation of brainstem regions involved in the arousal response, which in turn elicit listeners’ orienting behaviors that can be highly functional during predator encounters, and intra- and inter-group agonistic encounters [4]. However, this idea does not explain why acoustically similar calls (e.g., alarm and aggressive calls: [13]) can elicit different responses in conspecifics (e.g., escape responses or aggression: [14]). On the other hand, the “informational perspective” proposes that listeners integrate call perception (e.g., arousal level, caller identity) with contextual information (e.g., presence of another group) in order to produce context specific responses [15].

Vocal exchange is a phenomenon suitable to analyze the association between the acoustic variation of contact calls and listeners’ responses: it allows the proper identification of listeners (i.e., second callers), and two types of responses, the vocal and the behavioral (e.g., orienting behaviors: [16–18]). Among the nonhuman primate species that exchange calls (e.g., *Callithrix jacchus*: [16]; *Macaca fuscata*: [17]; *Cantorchilus zeledoni*: [18]), it is well accepted that spider monkeys (*Ateles geoffroyi*) exchange long-distance contact calls termed “whinnies” [9]. In our previous study, we found that whinnies have a graded acoustic structure, and a high amount of acoustic variation related to the caller’s immediate behavior (e.g., scanning, looking toward the observer) and separation distance between callers [19]. However, we did not assess: (i) whether the acoustic variation of spontaneous whinnies (i.e., whinnies that are not responses to other whinnies) is related to caller context (e.g., social isolation) and the likelihood of producing a response whinny; and (ii) whether whinny acoustic variation and/or separation distance trigger listeners’ responses (i.e., second callers) that can facilitate contact.

Here, we analyzed whether the acoustic variation of spontaneous whinnies is related to caller social isolation (i.e., inside or outside a subgroup) and the likelihood of producing a response whinny in free-ranging spider monkeys (*A*. *geoffroyi*). Moreover, we analyzed whether whinny acoustic variation affects listeners’ responses that can facilitate contact (i.e., short latency to respond, orienting behaviors [scanning or looking toward the previous caller], whinny acoustic variation). Since social isolation can induce an increase in arousal levels (heart rate) [7], and studies in spider monkeys suggest that an increase in the arousal level (e.g., physical aggression that involves physical contact: [20]; performing of immediate behaviors: [19]) decreases a whinnies’ frequency (Hz), we predicted that whinnies produced by callers outside a subgroup would have lower frequencies (Hz). Moreover, as studies suggest that arousing acoustic features can elicit responses in listeners [4, 12], we predicted that independent of caller social isolation, low frequency whinnies would be more likely to elicit a response. However, based on the “informational perspective” [15], we hypothesized that listeners would consider contextual information (e.g., presence of another party) to produce suitable responses that facilitate contact with callers. Specifically, since it should be more difficult to maintain contact with callers separated by longer distances, we predicted that when previous callers produced whinnies at farther distances, listeners would: (i) respond faster; (ii) respond with low frequency whinnies; and (iii) perform orienting behaviors.

## Materials and methods

### Ethics statements

This research was carried out according to the ethical and legal requirements of the Secretaría de Medio Ambiente y Recursos Naturales (SEMARNAT) of Mexico, and was authorized by permit number SGPA/DGVS/03785/16. This permission allowed us to work in the Lacandona rainforest, and the authorities of the ejido Reforma Agraria allowed us to work in their natural reserve. This study was purely observational and was noninvasive: observers were always at more than five meters from spider monkeys. It also followed the American Society of Primatologists’ Principles for the Ethical Treatment of Primates.

### Study site and study community

We conducted our study in an old growth forest tract of 1,125 ha located in the Lacandona rainforest, Mexico (16°15’37”N, 90°50’12”W). This area is a natural reserve that belongs to the ejido Reforma Agraria that has a high density of spider monkeys’ food resources [21]. The climate in this region is warm and humid, the mean annual rainfall ranges between 2,500 and 3,500 mm, and the mean annual temperature ranges between 24 and 26°C (Comisión Federal de Electricidad, Mexico City). Although rainfall is relatively constant throughout the year, there is a pronounced dry season between February and April.

We studied a group of 35 adult (27 females, and 8 males) black-handed spider monkeys from a community of at least 148 individuals. JDOG and a local field assistant have studied this community between 2012 and 2016, and it is well habituated to human presence. The 35 study subjects were identified using anatomical features such as body size, scars, spots, hair and skin pigmentation.

#### Subgroup definition

**Fig 1:**
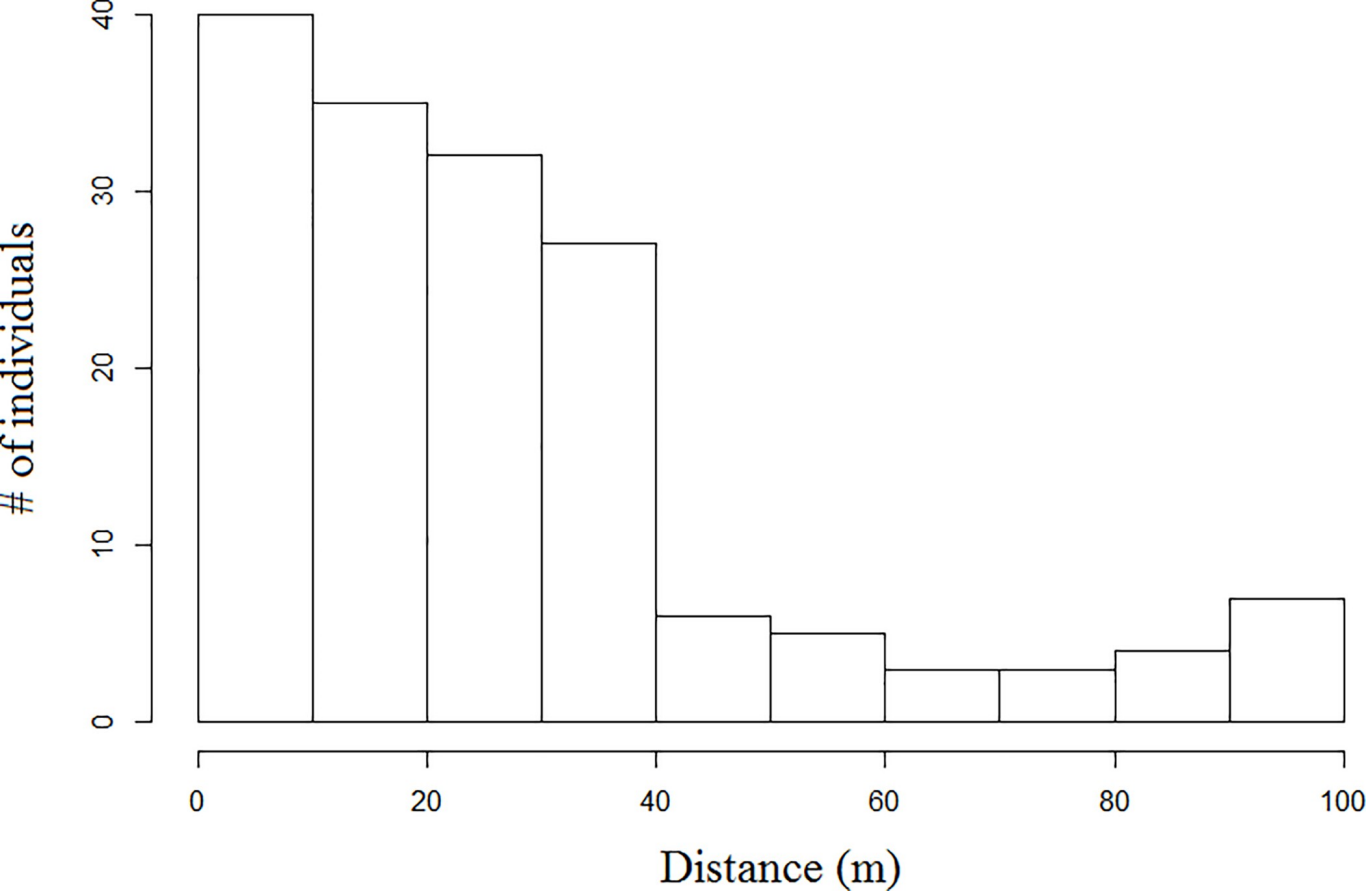
Histogram representing the distance distribution of spider monkeys (*Ateles geoffroyi*) located within a radius of 100 m from a focal subject, in the Lacandona rainforest, Mexico.

During December 2015 and January 2016 we followed the study group from 0600 to 1500 h for four days every week in order to determine the criterion to define a subgroup, re-identify group members, and locate their most frequent traveling routes. To select this criterion, we followed a similar method as that described by Ramos-Fernández [9]. First, we selected a focal adult spider monkey, and while one observer marked its location, two observers recorded via GPS (global positioning systems) the location of all the adult individuals that were within a radius of ≤ 100 m from the focal animal. We repeated this procedure 20 times during different days and with different focal individuals. Then, we created a histogram representing the distribution of individuals at the distances recorded. The histogram revealed an abrupt steep decline at 40 m (Fig 1), therefore, we classified the callers that were at ≤ 40 m from other adult individuals as callers that were inside a subgroup, and callers that were not surrounded by other adult individuals at ≤ 40 m, as callers that were outside a subgroup (i.e., isolated callers).

### Data collection

We conducted our data collection from February 2016 to June 2016 since most of this period coincides with the Lacandona rainforest dry season, and audio-recordings are difficult to collect during the rainy season. During this period, we (JDOG and a local field assistant) followed the spider monkeys from 0600 to 1600 h for four days every week. Each day we selected a spider monkey and used focal animal sampling [22] to record data when subjects were within 20 m from our microphones, since this distance is suitable for the analysis of whinny acoustic variation [19]. When we identified another individual that was at ≤ 20 m from microphones and at ≤ 60 m from the focal animal, we simultaneously sampled both individuals, and when we identified more than two individuals that fulfilled both criteria, we switched between them at 5-minute intervals. The highest number of individuals that we sampled at the same time was four; a focal animal that we selected each sampling day, and three that we switched between at 5-minute intervals. We followed the spider monkeys for a total of 680 h (range per subject: 14–21 h): Each study subject was followed for two consecutive days. We recorded the whinnies exchanged within the 60-m distance range, and although observers used short-wave radios (Motorola MJ270MR (Motorola Chicago, United States, Chicago, Illinois)) to stay in contact, visual contact facilitated observer coordination during simultaneous samplings. To record whinnies, each observer used a directional microphone (Sennheiser ME67 (Sennheiser Electronics, Wedemark, Germany)) and a digital audio recorder (Marantz PMD 661 (Marantz Europe, Eindhoven, Netherlands)) set at a sample rate of 44.1 kHz, a 16-bit resolution, and a mono format.

For each whinny recorded, we noted the identity of the caller, presence or absence of orienting behaviors, the vocal event (i.e., each whinny that was not produced during a vocal exchange or vocal exchanges comprised by several whinnies), the sampling day, the emission type and the separation distance between individuals that exchanged whinnies. Moreover, when we could identify if other adult individuals were at ≤ 40 m from callers, we also noted if the focal animal was inside or outside of a subgroup. To do this, ten seconds after recording each whinny, observers scanned if other spider monkeys were at ≤ 40 m from the focal animals, and we only noted this information when both observers agreed. We coded orienting behaviors as those that involve “scanning” or “looking toward the previous caller” during the whinny emission and up to the two seconds afterwards.

The procedure that we followed to identify if a whinny was produced during a vocal exchange, has been described elsewhere [19]. In sum, we identified the start of the whinnies produced by focal animals, and within an interval of ±10 s, we measured the times between the start of each whinny. We classified the whinnies produced within ±1 SD (±1.43 s) from the average (1.87 s) of these times as “whinnies produced during vocal exchanges”. This criterion is appropriate since the curve of the probability of producing a response whinny reveals a low amount of skew and the 92.8% of the whinnies produced within the 10 s interval were located within this range [19]. Regarding the emission type, we classified “spontaneous whinnies” as those that were produced outside of a vocal exchange, or the first whinny produced during a vocal exchange. “Response whinnies”, were those produced during vocal exchanges after the initial spontaneous whinny. We defined independent “vocal events” as each whinny that was not produced during a vocal exchange, or those produced during the same vocal exchange.

We classified whinnies into three separation distance categories and one “no vocal exchange” category: (i) 0–20 m; (ii) 21–40 m; (iii) 41–60 m; and (iv) no vocal exchange. We were not able to record whinnies exchanged at > 60 m for the reasons explained above, however, our data suggest that in our study site, whinny exchange occurs more frequently within 0–40 m [19]. It is important to clarify that we did not consider in the analyses those spontaneous whinnies that prompted response whinnies from listeners located at > 60 m from callers.

### Acoustic analyses

**Fig 2:**
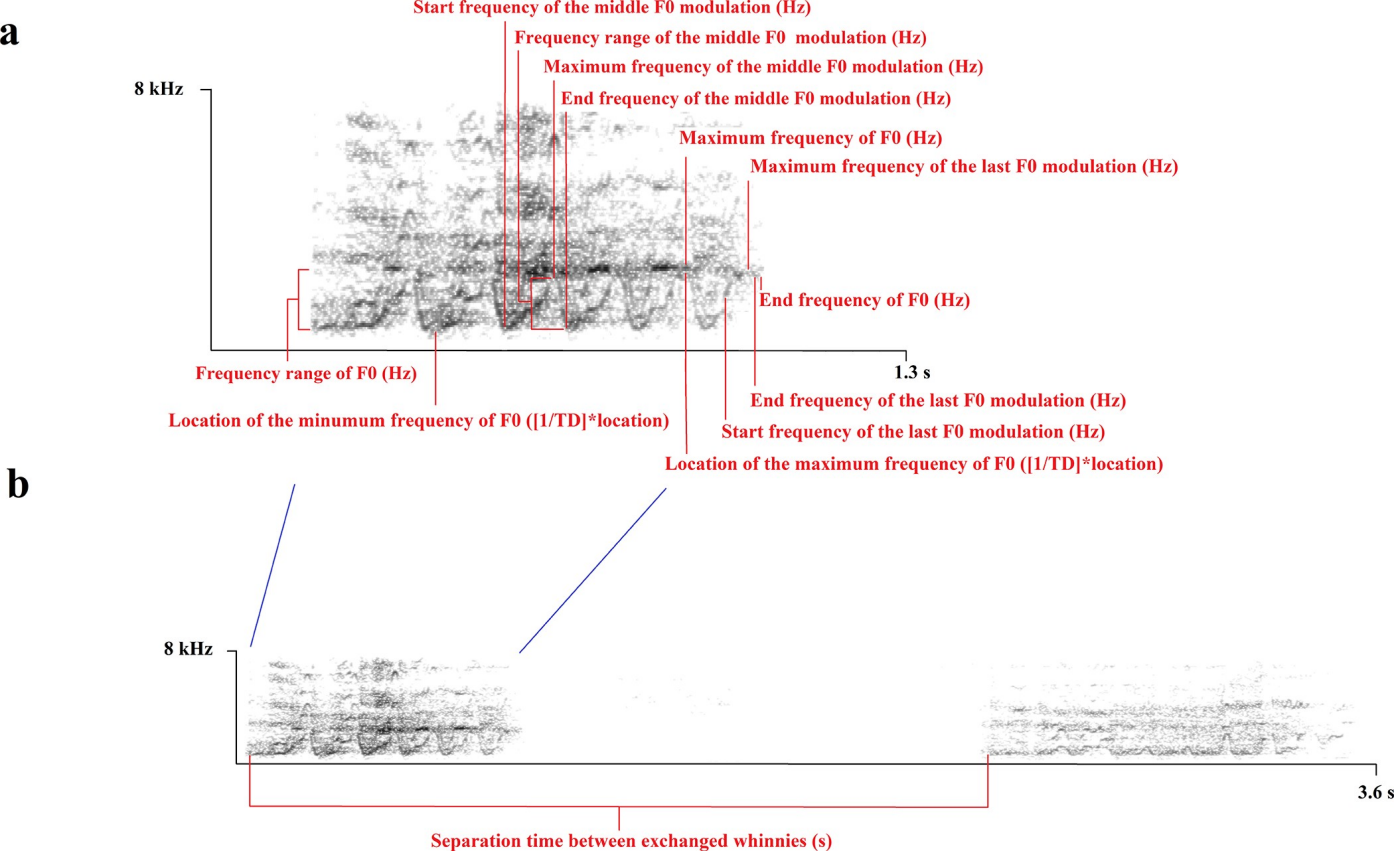
Whinnies produced by free-ranging spider monkeys (*Ateles geoffroyi*) in the Lacandona rainforest, Mexico. (a) Acoustic parameters that better explained the acoustic variation of 566 whinnies produced by 35 spider monkeys. (b) Separation time between exchanged whinnies.

Based on a previous definition [23], and on the acoustic variation of the whinnies that we recorded, we defined a whinny as a call with three or more rises and falls in the frequency domain (i.e., modulations) that can range between *ca*. 300 to 5,300 Hz, with a duration of *ca*. 0.4 to 3.5 s (Fig 2). In total, we analyzed the acoustic variation of 566 whinnies produced by 35 individuals (S1 Table), and extracted 42 acoustic parameters which were selected based on previous studies and acoustic qualities considered helpful to describe whinny variation [24–26] (S2 Table). To analyze acoustic parameters, we used Avisoft version 5.2.05 (Avisoft SASLab Pro) with a 16 kHz sampling frequency, a fast Fourier transform of 256 samples, and a Hamming window with an overlap of 96.9%. In addition, we measured the separation time between the starts of whinnies produced by focal animals, and the start of the whinnies produced by other animals within a ±10 s range (Fig 2B).

### Description of whinny acoustic variation

To describe whinny acoustic variation, we used an unsupervised classification procedure (i.e., 2-step cluster analyses) to avoid the use of subjective classifications (e.g., human visual classifications or predetermined classifications based on caller behaviors). To run these analyses, we used the 42 acoustic parameters and the first three factors calculated through a factor analysis using all the acoustic parameters. This procedure is described in Ordóñez-Gómez et al. [19]. Factor 1 was the variable that best described whinny acoustic variation, and represented 28.345% of the total variation. This factor was associated with ten frequency parameters that showed positive loadings, and only two temporal parameters that showed loadings with opposite patterns (i.e., parameters that showed loadings > 0.4 or < -0.4) (Fig 2A). Since all of the frequency parameters were positively associated with this factor, factor 1 was easy to interpret: an increase in factor 1 indicates an increase in F0 frequencies (Hz) [19].

### Statistical analyses

In total, we ran four mixed models using the lmer function of the lme4 package for R using different sets of whinnies. To test the collinearity among predictors, we calculated the variance inflation factor (VIF) for all models using the function vif of the car package for R. All models showed a VIF < 4, which indicates a low collinearity [27, 28]. In order to check the assumptions of homogeneity and normally distributed residuals, we visually inspected Q-Q plots of residuals plotted against fitted values, for the four LMMs that we ran. All analyses were carried out using RStudio 1.0.143 interface of R 3.1.1.

Model 1: To analyze the relationship between caller social isolation and whinny acoustic variation, we ran a LMM using 183 spontaneous whinnies produced by 23 callers from which we could determine if they were inside or outside a subgroup. This set of whinnies represented the 53.8% of the 340 spontaneous whinnies that we recorded. For this, we specified factor 1 as the response variable, since it allowed the best cluster classification, and therefore, the best description of whinny acoustic variation. We specified caller social isolation (inside or outside a subgroup) and response/no response as fixed factors, and caller identity as a control factor (i.e., random factor). Model 1.1: As the effect of caller social isolation on F1 could be potentially related to the separation distance between individuals that exchange calls, we tested using a single LMM the effect of these two variables on F1. For this model, we used the same data set of model 1 and we specified factor 1 as the response variable, separation distance and caller social isolation as fixed factors, and caller identity as a control factor.

Model 2: To analyze if the listener response time was related to the acoustic variation of the previous whinny and/or distance between callers, we ran a LMM using the information from 131 whinnies, from which we could determine the identity of both callers (i.e., previous (*N* = 23) and following (N = 23) callers). For this model, we specified response time between exchanged whinnies as the response variable, factor 1 and emission type of whinnies produced by previous callers (spontaneous or response whinnies) and separation distance as fixed factors, and identity of the previous and following caller, and vocal exchange, as control factors.

Model 3: To analyze whether the acoustic variation of whinnies produced by listeners (i.e., following callers) was related to the acoustic variation of whinnies produced by previous callers and/or separation distance, we ran a LMM using the information from 43 vocal exchanges from which we could analyze the acoustic variation of the whinnies exchanged and determine the identity of both callers (i.e., 18 previous callers and 18 following callers). In this model, we specified factor 1 of the whinnies produced by listeners as the response variable, factor 1 of the whinnies produced by previous callers, separation distance and emission type of the previous whinny as fixed factors, and the identity of both callers as control factors. In this model we did not control for vocal exchange, since all the whinny exchanges were produced during different vocal events.

Model 4: To identify whether the orienting behavior of listeners’ (i.e., following callers) was related to the acoustic variation of whinnies produced by previous callers and/or separation distance, we ran a GLMM with a binomial response variable using the information from 52 vocal exchanges from which we could analyze the acoustic variation of the whinny produced by the previous caller, determine listeners’ orienting behaviors, and both callers’ identities (i.e., 18 previous callers and 21 following callers). For this model, we specified presence or absence of listeners’ orienting behaviors as the response variable, factor 1 and emission type of whinnies produced by previous callers and separation distance as fixed factors, and the identity of both callers as control factors. Again, we did not control for vocal exchange, since all the whinny exchanges were produced during different vocal events.

For all of the models, we conducted *post hoc* pairwise comparisons between distance categories with the function lsmeans of the package lsmeans for R, with p values adjusted using the Tukey method.

## Results

**Fig 3:**
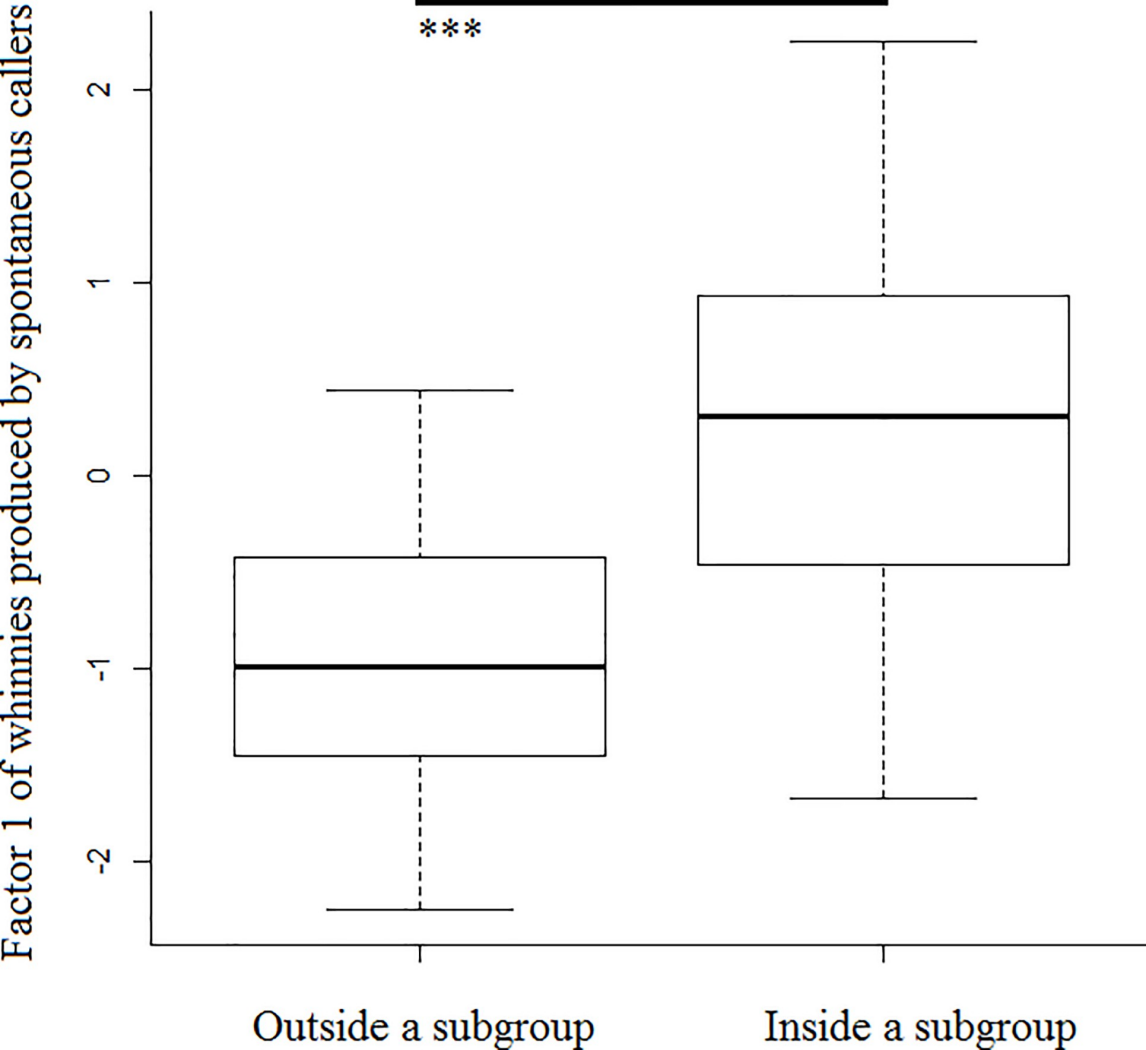
Box plots representing the distribution of factor 1 values across caller social isolation categories. ***Indicate significant differences (p < 0.001) between inside and outside subgroups.

**Fig 4:**
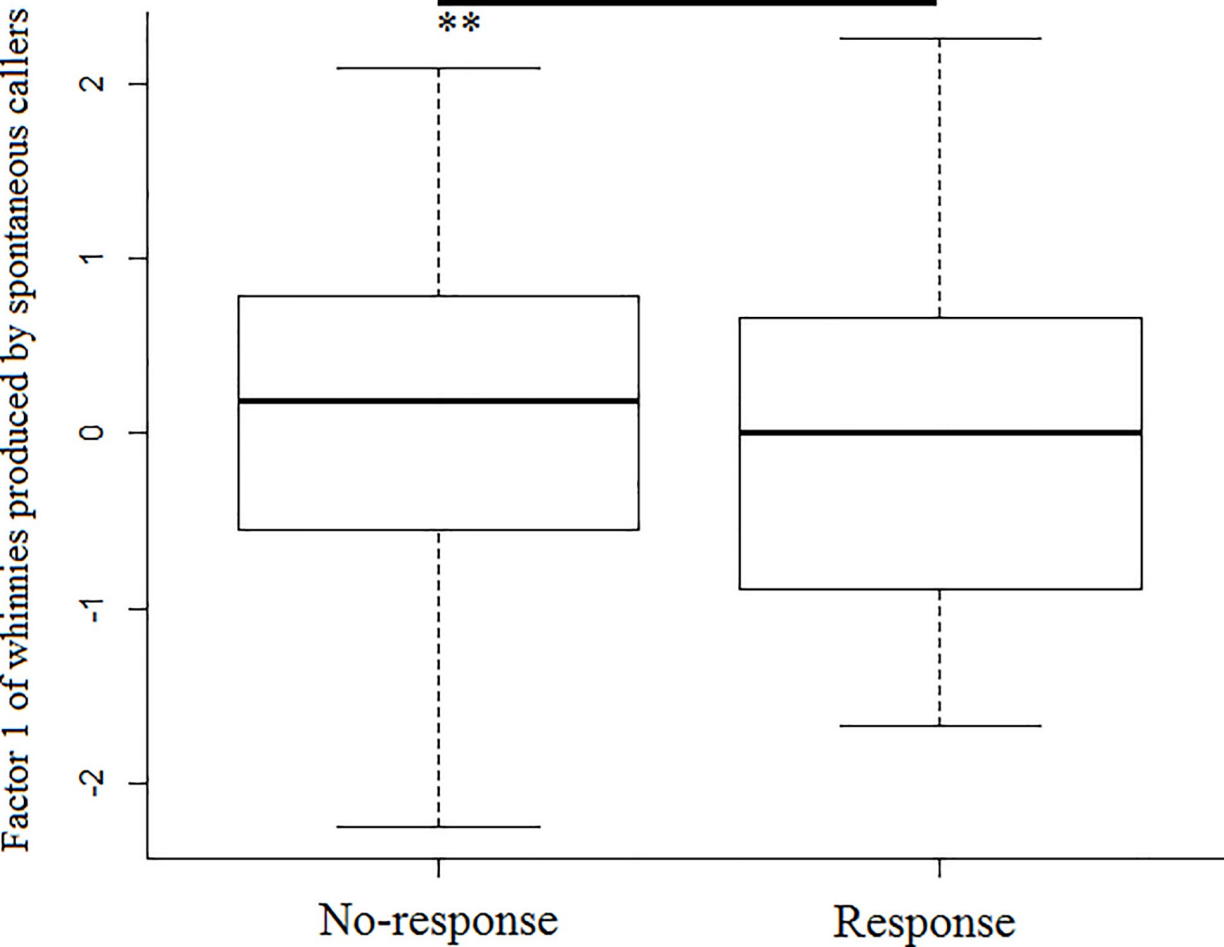
Box plots representing the distribution of factor 1 values across response categories. **Indicate significant differences (p < 0.01) between no-response and response.

**Fig 5:**
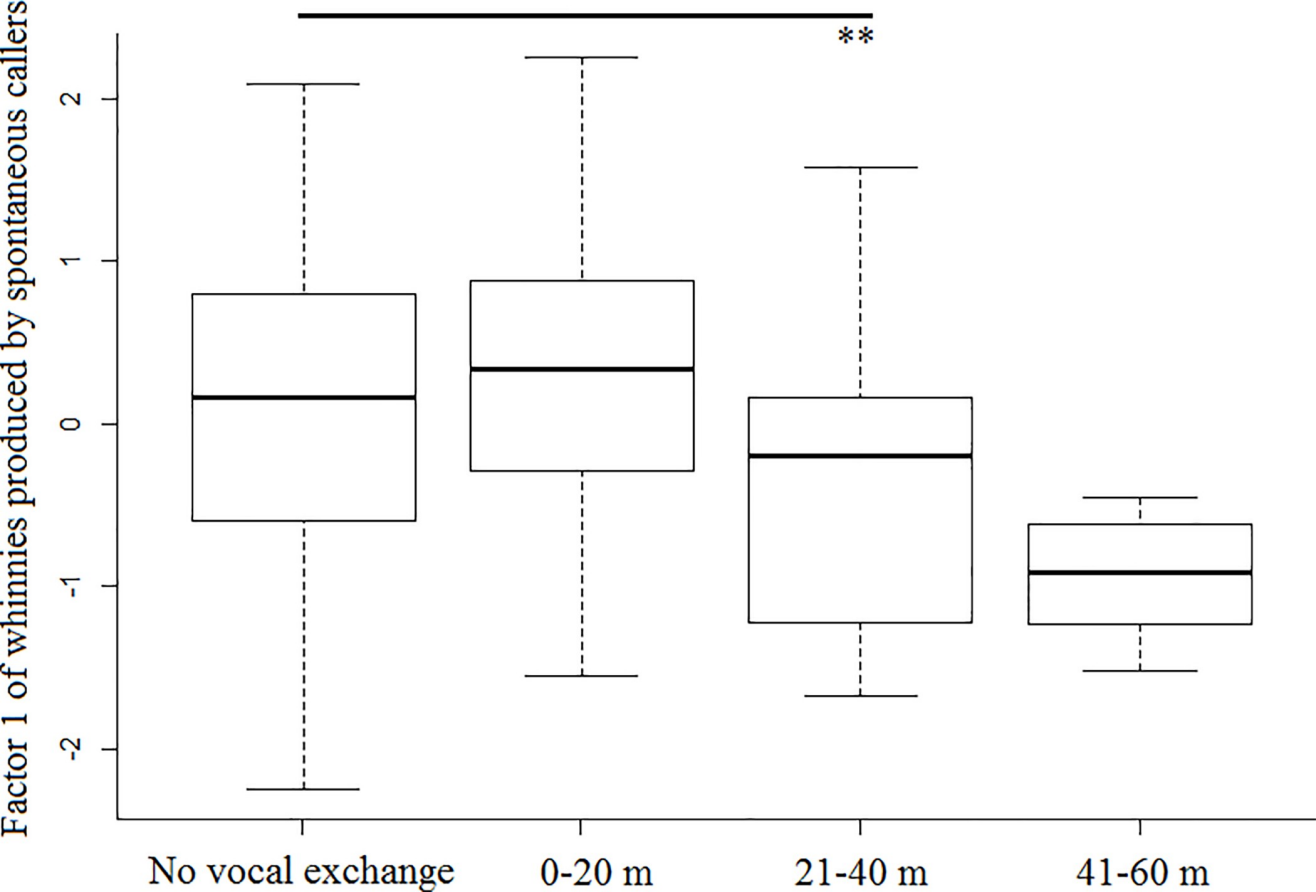
Box plots representing the distribution of factor 1 values across distance categories. **Indicate significant differences (p < 0.01) between no-response and response.

**Fig 6:**
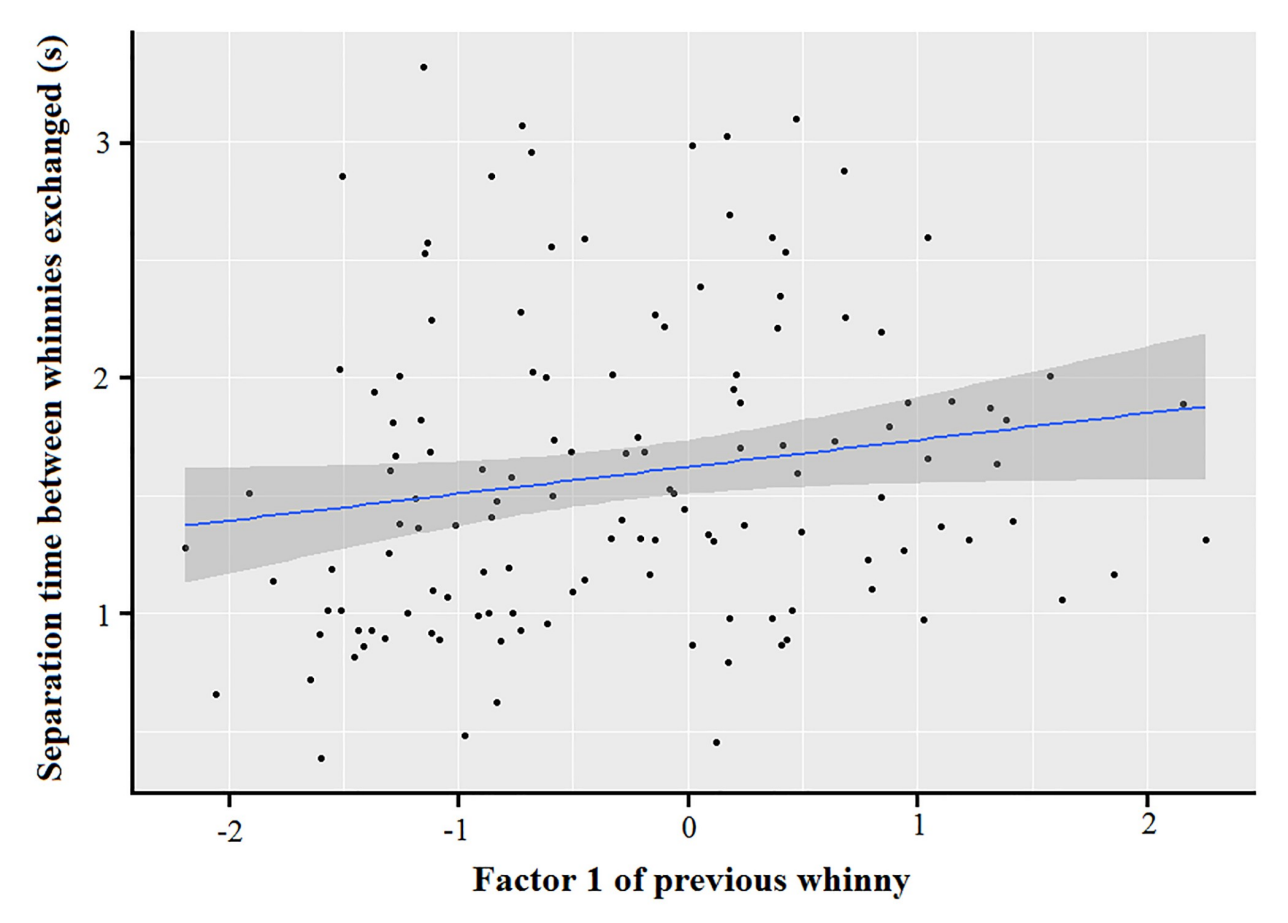
Association between separation time of whinnies produced during vocal exchanges and factor 1. The 0.95 confidence level is indicated in gray.

**Fig 7:**
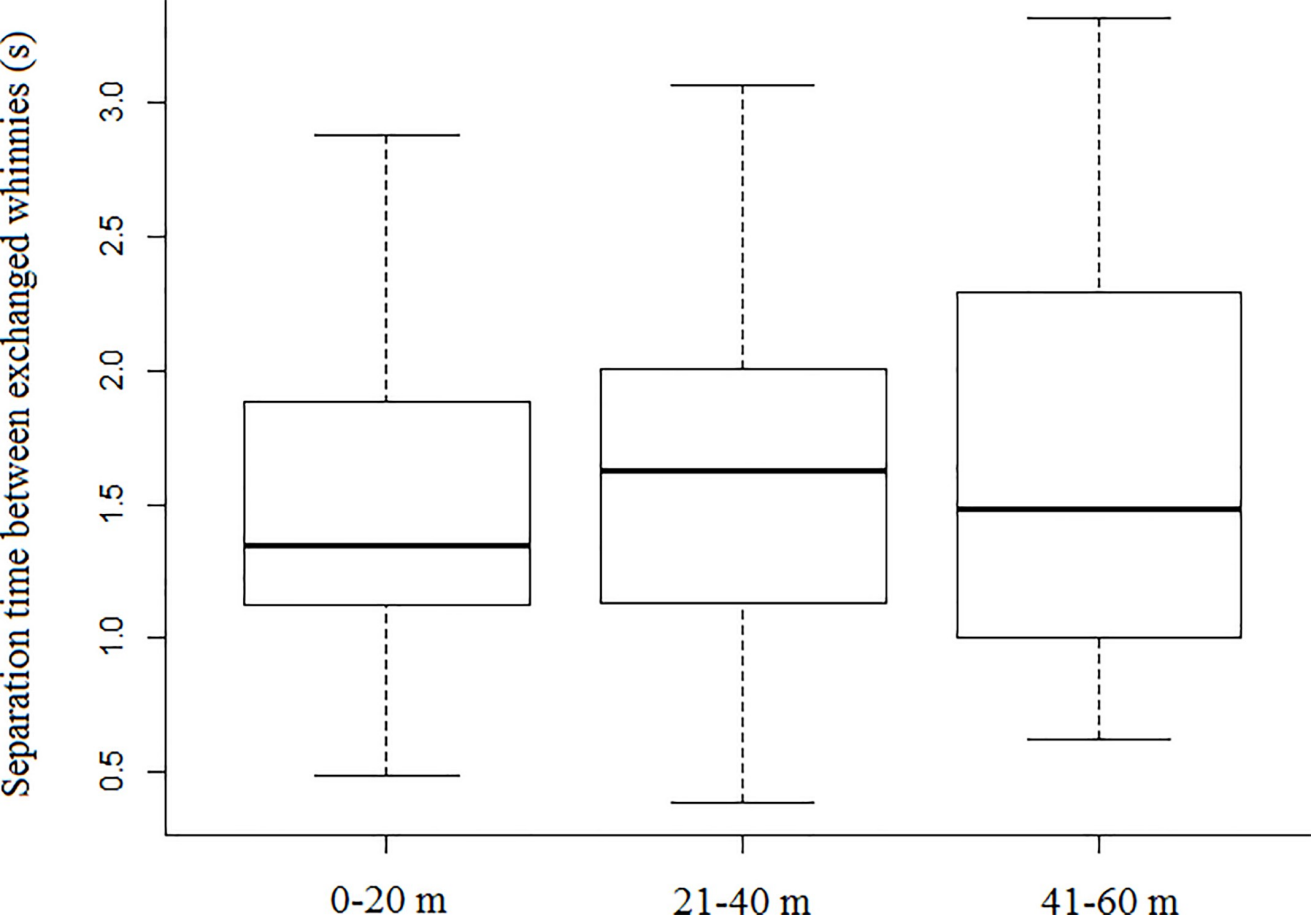
Box plots representing the distribution of separation times between whinnies produced during vocal exchanges, across distance categories.

**Fig 8:**
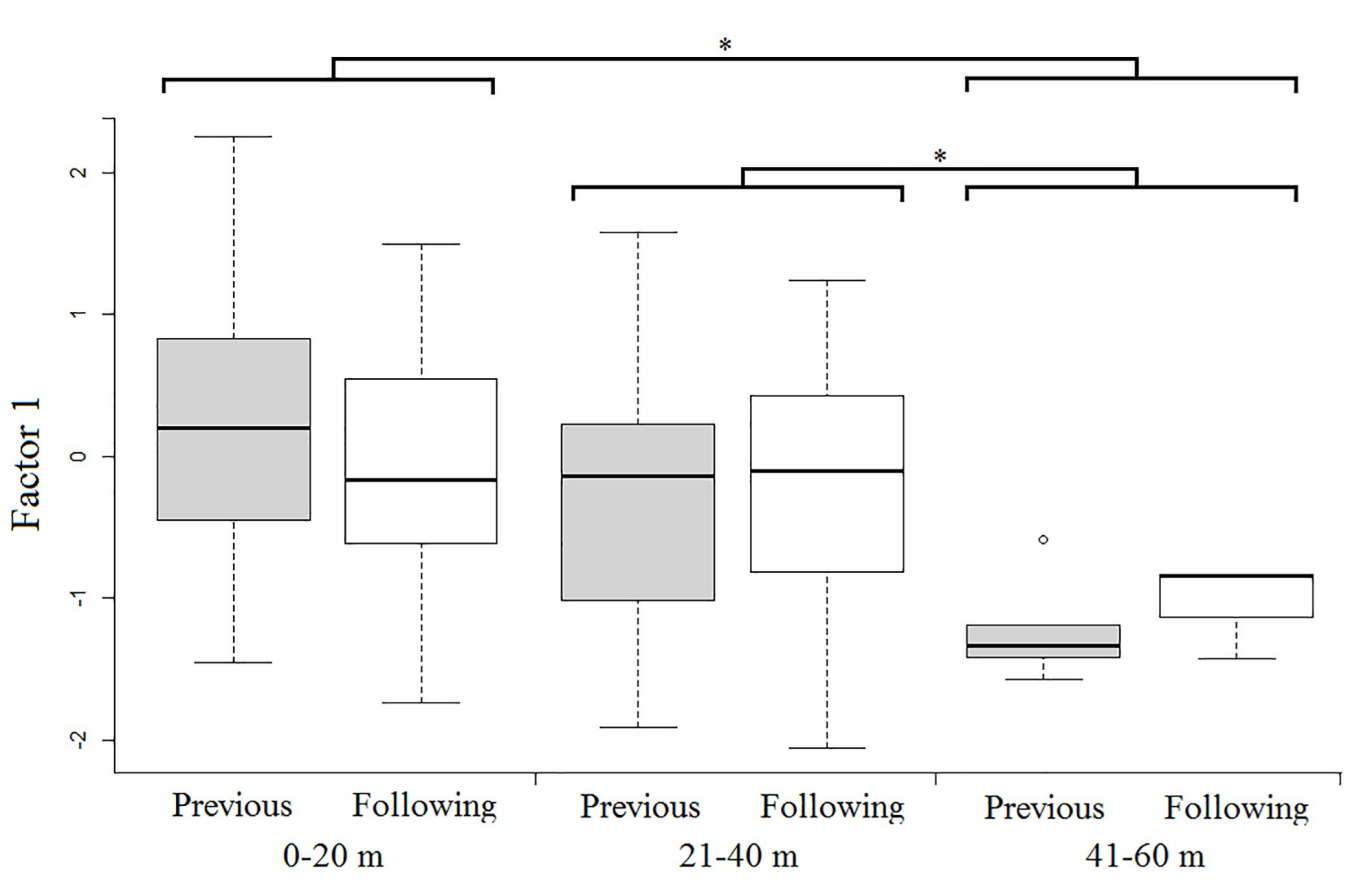
Box plots representing the distribution of factor 1 values across distance categories and across callers that exchanged whinnies. *Indicate significant differences (p < 0.05) between distance categories.

Model 1 revealed that spontaneous callers that were outside a subgroup produced whinnies with lower frequencies (i.e., lower factor 1 values), and showed that lower frequencies are more likely to elicit a response (Table 1; Figs 3 and 4). Model 1.1 confirmed the effect of caller social isolation, and that whinnies exchanged by callers separated by longer distances have lower frequencies (Table 1; Figs 3 and 5). Model 2 revealed that response time was positively related to frequency values of previous whinnies and separation distance between callers (Table 1; Fig 6), however, post hoc pair wise comparisons did not reveal significant differences among distance categories (Fig 7). Model 3 showed that frequencies of response whinnies were not related to frequencies of previous whinnies, but were related to the separation distance between callers (Table 1). In sum, listeners respond with lower frequency whinnies to callers separated by longer distances (Fig 8). Finally, model 4 showed that the performance of listeners’ orienting behaviors was not related to acoustic variation of previous whinnies nor separation distance between callers (Table 1).

**Table 1:** Results of four different models examining the effect of caller social isolation on whinny acoustic variation (Factor 1 variation), and the factors influencing listeners’ responses (i.e., following callers).

Model/Response variable~Predictor variables	χ^2^	d. f.	p
**Model 1/Factor 1~Caller social isolation+Respond**			
Caller social isolation	61.413	1	0.000
Respond	10.736	1	0.001
** Model 1.1/Factor 1~Caller social isolation+Separation distance**			
Caller social isolation	50.867	1	0.000
Separation distance	18.543	3	0.000
**Model 2/Response time~Factor 1 (previous whinny)+Separation distance+Emission type**			
Factor 1	10.709	1	0.001
Separation distance	6.004	2	0.049
Emission type	0.642	1	0.423
**Model 3/Factor 1 (response whinny)~Factor 1 (previous whinny)+Separation distance+Emission type**			
Factor 1	0.179	1	0.672
Separation distance	8.587	2	0.014
Emission type	0.690	1	0.406
**Model 4/Orienting behaviors (listeners)~Factor 1 (previous whinny)+Separation distance+Emission type**			
Factor 1	1.240	1	0.265
Separation distance	2.940	2	0.223
Emission type	0.283	1	0.595

Factor 1 is positively associated with 10 frequency parameters of F0 and two temporal parameters that showed opposite patterns (i.e., parameters that showed loadings > 0.4 or < -0.4).

## Discussion

Our study revealed that spontaneous callers outside a subgroup produced whinnies with lower frequencies than those produced by spontaneous callers inside a subgroup, and that listeners’ responses were related to both the acoustic variation of the previous whinny and separation distance between callers. Listeners had a faster response to lower frequency whinnies, and produced lower frequency whinnies when there was a greater separation distance between callers. These findings reveal that listeners’ responses are related to call acoustic variation and contextual information (i.e., separation distance between callers), and therefore, support the “informational perspective” [15].

Given that spontaneous callers produced low frequency whinnies when they were not forming part of a subgroup, and it is well accepted that low frequency calls travel longer distances (e.g., [29]), our results suggest that whinny acoustic variation facilitates vocal contact under circumstances in which potential listeners are farther away. This suggests that cognitive representations that elicit different affective or motivational states could drive call acoustic variation into different directions [13]. Spider monkeys that are outside a subgroup could have a higher urgency of establishing contact with other group members, and therefore, a higher arousal level. Supporting this notion, previous studies conducted on spider monkeys revealed that agonistic vocalizations show a frequency decrease when the agonistic encounter involves physical contact [20], and whinnies have a lower F0 when callers perform immediate behaviors (i.e., rapid reactions) [19]. This phenomenon can be explained if we consider that when arousal level increases, subglottal pressure increases, which in turn leads to a decrease of F0 due to irregular vibrations in the vocal folds [30, 31]. However, the negative relationship between arousal level and whinny frequency (Hz) is opposite to what has been found for other mammals’ calls [7, 32, 33]. Hence, modeling spider monkeys’ vocal tract configurations (e.g., vocal folds tension: [34]) during the production of whinnies, and analyzing the relationship between a proxy of arousal level (e.g., breathing frequency) and whinnies’ acoustic variation, would help to identify whether an increase in caller’s arousal level is negatively related to whinny frequencies (Hz) [7, 35].

Moreover, low frequency (Hz) whinnies were more likely to elicit a response, and with a shorter latency (independently of the separation distance between callers). This suggests that a frequency (Hz) decrease could trigger an increase in listener arousal levels, and supports that some acoustic qualities can work as arousing triggers (e.g., wiener entropy) that elicit a listener’s response [4, 12]. However, to better support this relationship it is also important to measure a well-accepted proxy of arousal level (e.g., heart rate), and assess whether it is related to whinny acoustic variation and listeners’ responses. It is important to mention that the null effect of separation distance on response time might not affect vocal contact, since listeners respond in very short intervals (between 0.44 and 3.30 s) to previous callers [19].

The fact that the acoustic structure of whinnies produced by listeners (i.e., following callers) was affected by the separation distance between callers, but not by the acoustic variation of the previous whinny, supports the “informational perspective”. Listeners seem to produce responses that are linked to both the acoustic variation of the previous whinny (i.e., likelihood of producing a response whinny and response time) and contextual information (i.e., separation distance between callers). This would be particularly important to the maintenance of group cohesion, since it would not make sense to respond with high frequency whinnies that travel short distances to callers that are separated by greater distances. Contrary to our prediction, we did not find a significant effect of separation distance nor whinny acoustic variation on the occurrence of listeners’ orienting behaviors. Listeners could be integrating other types of potential information that indicates caller location, such as previous interactions with callers, or call attenuation (e.g., [36, 37]). It is important to indicate that although we did not find a significant effect of whinny acoustic structure and context on the acoustic structure of whinnies produced by listeners and their orienting behaviors, these results should be carefully considered since the number of vocal exchanges included in these analyses was relatively small to fully support these conclusions.

In sum, our study shows that social isolation influences the acoustic variation of whinnies produced by spontaneous callers, and suggests that listeners integrate call perception (i.e., caller’s arousal level) and contextual information (i.e., separation distance to previous caller) in order to produce responses that facilitate vocal contact with previous callers. However, as we mentioned above, future research that assesses relationships between proxies of arousal level and whinny variation, and between callers’ association index and listeners’ responses, would provide stronger support for this claim. Nevertheless, the fact that spider monkeys live in dense forests and fission-fusion societies generates contexts of high social uncertainty [38]. Hence, the production of low frequency whinnies when callers are outside a subgroup, and respond with low frequency whinnies to callers separated by long distances, could be of high importance to facilitate contact with out-of-sight group members, and therefore to reduce social uncertainty.

## Supporting information

S1 Table Distribution of 566 whinnies produced by 35 free-ranging spider monkeys (*Ateles geoffroyi*) living in the Lacandona rainforest, Mexico. (DOCX)

S2 Table Acoustic parameters measured in 566 whinnies produced by 35 free-ranging spider monkeys (*Ateles geoffroyi*) living in the Lacandona rainforest, Mexico. (DOCX)

S1 Sound file Whinny that did not produce a listener’s response. (WAV)

S2 Sound file Whinny that produced a response from a listener located in the caller’s group. (WAV)

S3 Sound file Whinny that produced a response from a listener that was not located in the caller’s group. (WAV)

S1 Dataset Sets of data used to run the models. (XLSX)
