# Supplementary material for: Acoustic variation of spider monkey (Ateles geoffroyi) contact calls is related to caller isolation and affects listeners’ responses
Source: PLoS One. 2019 Apr 3;14(4):e0213914. doi: 10.1371/journal.pone.0213914 (PMC6447145; doi:10.1371/journal.pone.0213914)
Supplement: S1 Table — (DOCX) [file pone.0213914.s001.docx]

**S1 Table. Distribution of 566 whinnies produced by 35 free-ranging spider monkeys (*Ateles geoffroyi*) living in the Lacandona rainforest, Mexico.**

| **Subject code** | **Sex** | **Total** |
| --- | --- | --- |
| AE | F | 23 |
| AM | M | 1 |
| BE | M | 3 |
| CA | F | 64 |
| FE | F | 2 |
| FR | F | 50 |
| FRI | F | 20 |
| FRT | F | 8 |
| GE | M | 1 |
| GL | M | 1 |
| H1 | F | 1 |
| HB | M | 25 |
| HD | F | 12 |
| HO | M | 1 |
| IR | F | 2 |
| JM | F | 5 |
| KA | F | 9 |
| LO | F | 1 |
| LU | F | 67 |
| MA | F | 20 |
| MD | M | 4 |
| MI | F | 47 |
| MP | F | 9 |
| MT | F | 53 |
| MU | F | 41 |
| NA | F | 2 |
| NP | F | 3 |
| PE | F | 4 |
| PI | F | 15 |
| RA | F | 21 |
| RO | F | 20 |
| TO | M | 7 |
| VE | F | 20 |
| VI | F | 1 |
| VR | F | 3 |
| Total | 148 | 566 |

F = females; M = males.
