# Supplementary material for: Acoustic variation of spider monkey (Ateles geoffroyi) contact calls is related to caller isolation and affects listeners’ responses
Source: PLoS One. 2019 Apr 3;14(4):e0213914. doi: 10.1371/journal.pone.0213914 (PMC6447145; doi:10.1371/journal.pone.0213914)
Supplement: S2 Table — (DOCX) [file pone.0213914.s002.docx]

**S2 Table. Acoustic parameters measured in 566 whinnies produced by 35 free-ranging spider monkeys (*Ateles geoffroyi*) living in the Lacandona rainforest, Mexico.**

| **Codes** | **Definitions** |
| --- | --- |
| TD | Total duration (s) |
| SF1stNEB1^st^F0M | Start frequency of the first no-modulated element situated before the first F0 modulation (Hz) |
| D1^st^NEB1^st^F0M | Duration of the 1^st^ no-modulated element situated before the first F0 modulation (s) |
| SFF1^st^F0M | Start frequency of the 1^st^ F0 modulation (Hz) |
| LSF1^st^F0M | Location of the start frequency of the 1^st^ F0 modulation ([1/TD]*location) |
| MF1^st^F0M | Maximum frequency of the 1^st^ F0 modulation (Hz) |
| LMF1^st^F0M | Location of the maximum frequency of the 1^st^ F0 modulation ([1/TD]*location) |
| EF1^st^F0M | End frequency of the 1^st^ F0 modulation (Hz) |
| LEF1^st^F0M | Location of the end frequency of the 1^st^ F0 modulation ([1/TD]*location) |
| TBSMF1^st^F0M | Time between the start and the maximum frequency of the 1^st^ F0 modulation (s) |
| TBMEF1^st^F0M | Time between the maximum and the end frequency of the 1^st^ F0 modulation (s) |
| D1^st^F0M | Duration of the 1^st^ F0 modulation (s) |
| FR1^st^F0M | Frequency range of the 1^st^ F0 modulation (Hz) |
| TB1^st^2^nd^F0M | Time between the 1^st^ and the 2^nd^ F0 modulation (s) |
| SFMF0M | Start Frequency of the middle F0 modulation (Hz) |
| LSFMF0M | Location of the start frequency of the middle F0 modulation ([1/TD]*location) |
| MFMF0M | Maximum frequency of the middle F0 modulation (Hz) |
| LMFMF0M | Location of the maximum frequency of the middle F0 modulation ([1/TD]*location) |
| EFMF0M | End frequency of the middle F0 modulation (Hz) |
| LEFMF0M | Location of the end frequency of the middle F0 modulation ([1/TD]*location) |
| TBSMFMF0M | Time between the start and the maximum frequency of the middle F0 modulation (s) |
| TBMEFMF0M | Time between the maximum and the end frequency of the middle F0 modulation (s) |
| DMF0M | Duration of the middle F0 modulation (s) |
| FRMF0M | Frequency range of the middle F0 modulation (Hz) |
| DBMNF0M | Duration between the middle and the next F0 modulation (s) |
| SFLF0M | Start frequency of the last F0 modulation (Hz) |
| LSFLF0M | Location of the start frequency of the last F0 modulation ([1/TD]*location) |
| MFLF0M | Maximum frequency of the last F0 modulation (Hz) |
| LMFLF0M | Location of the maximum frequency of the last F0 modulation ([1/TD]*location) |
| EFLF0M | End frequency of the last F0 modulation (Hz) |
| LEFLF0M | Location of the end frequency of the last F0 modulation ([1/TD]*location) |
| TBSMFLF0M | Time between the start and the maximum frequency of the last F0 modulation (s) |
| TBMEFLF0M | Time between the maximum and the end frequency of the last F0 modulation (s) |
| DLF0M | Duration of the last F0 modulation (s) |
| FRLF0M | Frequency range of the last F0 modulation (Hz) |
| MAXFF0^a^ | Maximum frequency of F0 (Hz) |
| LMAXFF0 | Location of the maximum frequency of F0 ([1/TD]*location) |
| MINFF0 | Minimum frequency of F0 (Hz) |
| LMINFF0 | Location of the minimum frequency of F0 ([1/TD]*location) |
| EFF0 | End frequency of F0 (Hz) |
| FRF0 | Frequency range of F0 (Hz) |
| RF0MP | Rate of F0 modulation production (# of modulations/TD) |
